# Late-Onset Leigh Syndrome With Protracted Gastrointestinal Manifestations: A Rare Case Report

**DOI:** 10.7759/cureus.59669

**Published:** 2024-05-05

**Authors:** Zain Amar, Helai Hussaini, Meet Popatbhai Kachhadia, Iqra Samreen, Alaa S Mohamed, Hira Nasir

**Affiliations:** 1 Internal Medicine, Faculty of Medicine and Allied Medical Sciences, Hyderabad, PAK; 2 Internal Medicine, West Anaheim Medical Center, Anaheim, USA; 3 Internal Medicine, Pandit Deendayal Upadhyay Medical College (PDU) Civil Hospital Campus, Rajkot, IND; 4 Internal Medicine, Deccan College of Medical Sciences, Hyderabad, IND; 5 Neurology, Augusta University, Augusta, USA; 6 Internal Medicine, Mayo Hospital, Lahore, PAK

**Keywords:** late-onset leigh syndrome, neurodegenrative disease, adult-onset leigh syndrome, mitochondrial syndrome, leigh syndrome

## Abstract

Although Leigh syndrome (LS) is a neurodegenerative disorder of infancy, adult-onset LS has also been rarely reported. We report a case of late-onset LS in a 42-year-old female who presented with protracted gastrointestinal manifestations, chronic headaches, ataxia, and loss of consciousness. Brain magnetic resonance imaging (MRI) revealed hyperintensities in the bilateral basal ganglia and brain stem. Serum and cerebrospinal fluid lactate levels were significantly raised. Muscle biopsy showed reduced cytochrome oxidase (COX) activity. She was diagnosed with probable diagnosis of late-onset LS based on her clinical features, radiological findings, biochemical results, and biopsy findings. She responded well to intravenous thiamine, and her symptoms gradually improved.

## Introduction

Leigh syndrome (LS) is an uncommon neurogenerative disorder of infancy, characterized by subacute necrotizing encephalopathy and bilateral symmetrical gray matter necrotizing lesions in the basal ganglia, brain stem, and cerebellum [1]. LS typically presents in infancy, with over 50% of cases in the first year of life, mainly before six months of age, with a low prevalence of 1:40,000 births [2]. LS is a mitochondrial disorder that may be sporadic or familial, characterized by psychomotor regression or delay with lesions mainly in the brain stem and basal ganglia [3]. Adult-onset LS, also called late-onset LS, has been underlined in the literature, with only a few cases published. In adult-onset LS, symptoms may appear after two years and not manifest until early adulthood. Compared to the infantile form, adult-onset LS progresses slowly and is more prevalent in males [4,5]. Herein, we report a case of adult-onset LS in a female who presented with protracted gastrointestinal manifestations.

## Case presentation

A 43-year-old female presented with protracted generalized abdominal pain and intermittent vomiting for the last two months, for which she used over-the-counter medications with no improvements. She also complained of multiple episodes of throbbing headaches in the last year and was treated as migraine. Now, she presented with a worsening headache for the last week, which was generalized with no aggravating and relieving factors associated with bilateral ptosis and lightheadedness. She reported no history of febrile illness, seizures, limb weakness, sensory disturbance, or other preceding illnesses. She had no personal or family history of any similar disease. She reported no history of smoking or substance abuse but sporadic alcohol use.

She was hemodynamically stable and well-oriented to time, place, and person on examination. Eye examination revealed bilateral ptosis with horizontal gaze palsy and normal pupil. Fundus examination was unremarkable, with no signs of meningeal irritation. Cranial nerve examination was normal, and neurological examination showed ataxia. The rest of the systemic examination was unremarkable.

She underwent brain magnetic resonance imaging (MRI), revealing hyperintense signals in a widespread brain area. Electroencephalography (EEG) showed generalized slowing with no epileptiform activity. Owing to her protracted vomiting and MRI findings, she was diagnosed with a probable diagnosis of Wernicke’s encephalopathy. She was managed with parenteral thiamine, showed marked improvements in her symptomology, and was discharged.

**Figure 1:**
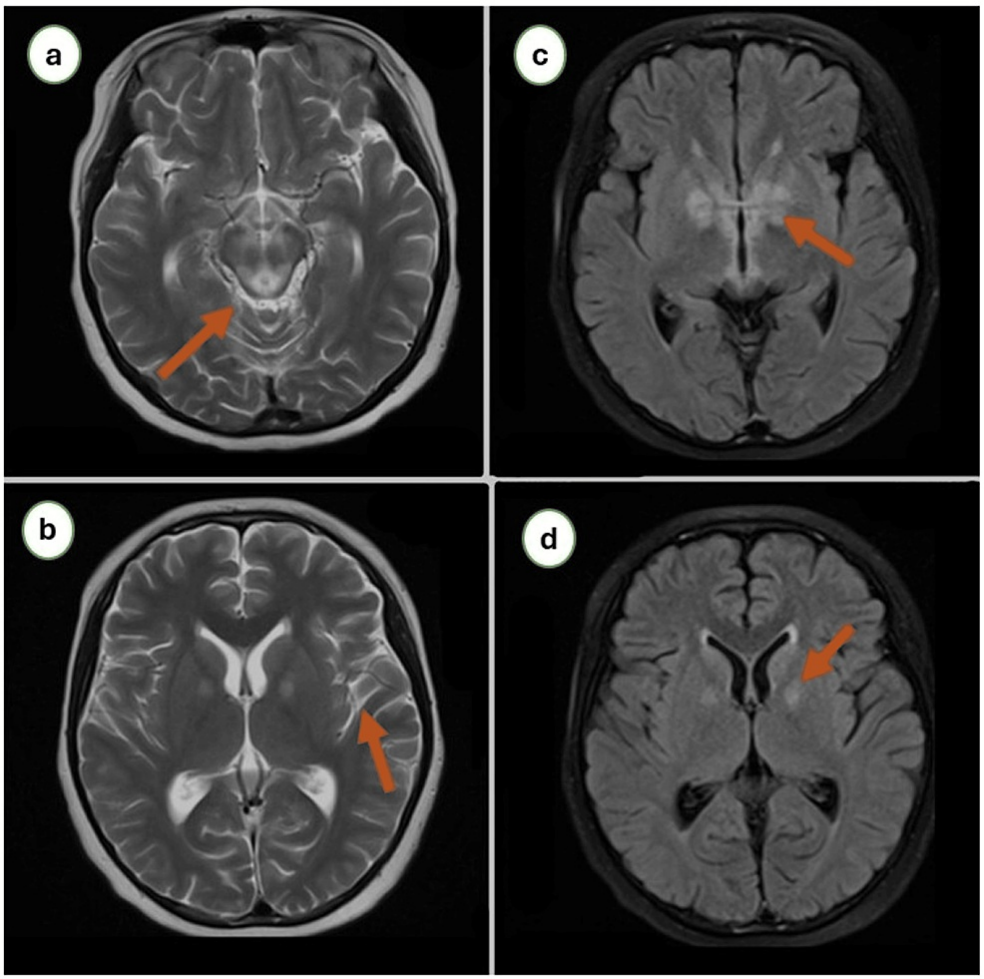
Brain MRI with T2-weighted (a,b) and diffusion-weighted (c,d) images showing marked bilateral signal abnormalities in the basal ganglia, brain stem, and brain white matter.

**Figure 2:**
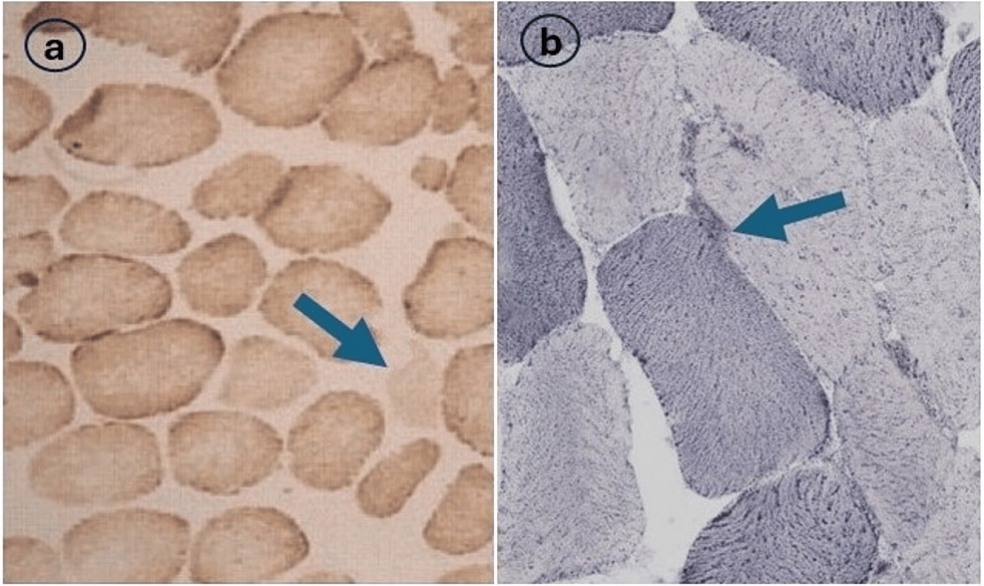
Histochemistry from muscle fiber showing reduced cytochrome oxidase activity (a) and subsarcolemmal aggregations (b). Stains: Hematoxylin and eosin. Magnification: 160x (a), 240x (b).

Two weeks later, she presented again with a severe headache followed by a loss of consciousness. On examination, she had brisk tendon reflexes and extensor plantar reflexes bilaterally. Her lab results were normal except for elevated fasting serum lactate level (Table 1). Arterial blood gas analysis revealed severe metabolic acidosis with a pH of 7.12. A repeat brain MRI showed hyperintense signals in bilateral basal ganglia, brain stem, and white matter (Figure 1). She underwent cerebrospinal fluid (CSF) analysis revealing an elevated lactate level of 3.9 mmol/L (normal: 1.1-2.3). The rest of the CSF biochemistry and cytology was within the normal range. Her blood culture was negative for any organism. She underwent detailed metabolic evaluations, including coagulation profile, serum copper, ceruloplasmin, and urinary copper, which were within normal range. Based on her clinical history and imaging and laboratory evaluations, a provisional diagnosis of adult-onset LS was made. She was managed with intravenous thiamine (300 mgm/day) with tapering dose, riboflavin, and co-enzyme-Q. Muscle biopsy from the quadricep muscle exhibited reduced cytochrome oxidase (COX) activity (Figure 2). Her muscle deoxyribonucleic acid (DNA) was extracted and analyzed for a complete mitochondrial genome, which showed no pathognomonic mutations.

**Table 1:** Laboratory findings on hospital admission.

Parameter	Lab value	Reference value
Hemoglobin	12.5	13-15 g/dl
White cell count	8700	4000-11000/mm^3^
Red cell count	4.3	4.2-5.2 million cells/mcl
Alanine aminotransferase	42	10-55 IU/L
Aspartate aminotransferase	36	8-39 IU/l
Serum creatinine	0.9	0.7-1.3 mg/dl
Platelet count	210,000	150,000-350,000/mcl
Blood urea nitrogen	22	7-28 mg/dl
Calcium	8.9	8.5-10.5 mg/dl
C-reactive protein	1.1	0.4-1.2 mg/dl
Serum lactate	8.1	0.2-2.4 mmol/l
Sodium	138	138-145 mEq/l
Potassium	4.1	3.5-4.5 mEq/l
pH	7.12	7.35-7.45

She improved gradually and became conscious on day five of admission, with a gradual improvement in headache and ptosis. Her gaze improved over one and a half months, and she started ambulating over the next two months. She remained asymptomatic over subsequent follow-ups.

## Discussion

LS is a progressive neurodegenerative disease of infancy and represents the most common pediatric clinical manifestation of mitochondrial disease [3]. Patients with LS typically present with episodic neurodegeneration, often leading to death at the age of three years. Although rare, adult-onset LS has also been reported, with only a few cases published. We have tabulated the reported cases of late-onset LS with clinicopathological presentation in Table 2 [4-10].

**Table 2:** Reported cases of late-onset Leigh syndrome. M: male, F: female, NR: not reported, MRI: magnetic resonance imaging, COX: cytochrome oxidase, CNS: central nervous system. Sources: [4-10]

Author et al.	Age/sex	Family history	Birth history	Presenting symptom	Organ involvement	Serum lactate level (mmol/L)	Muscle biopsy obtained	Mitochondrial gene mutation	MRI findings	Outcome
Goldenberg et al. [4]	24/F	No	Normal	Headache	CNS, respiratory	8	Yes, COX-negative	Yes	Brain stem hyperintensity	Regression
Cipriano et al. [5]	36/M	No	Normal	Walking difficulty	CNS, respiratory	7.8	Yes, COX-negative	No	Brain stem, basal ganglia hyperintensity	Regression
Wesolowska et al. [6]	45/M	No	Normal	History of fall	CNS, respiratory	3.7	Yes, reduced COX activity	No	Brain stem, basal ganglia hyperintensity	Ventilatory support
Lekha et al. [7]	26/M	No	Normal	Chronic headache	CNS	6.8	Yes, reduced COX activity	No	Midbrain, basal ganglia, grey matter hyperintensity	Regression
Liang et al. [8]	12/M	No	Normal	Blepharoptosis	CNS	4.4	NR	No	White matter, basal ganglia, midbrain hyperintensity	Died
Malogcic et al. [9]	21/F	Down syndrome	Normal	Headache, vomiting	CNS	11.9	Subsarcolemmal aggregations	Yes	Basal ganglia hyperintensity	Regression
Li et al. [10]	24/F	No	Normal	Seizure	CNS	NR	Subsarclolemmal aggregations	Yes	Basal ganglia, white matter hyperintensity	Died

LS has remarkable clinical and genetic heterogeneity; patients with LS typically present with characteristic neuropathological features. The characteristic manifestations of LS encompass psychomotor regression or delay, limb weakness, generalized hypotonia, tremors, and lactic acidosis detected in the CSF, blood, or urine [7]. According to published reports, typical manifestations of LS may also manifest in adult-onset LS. Patients with LS manifest headaches, dementia, intellectual decline, and vertical gaze palsy based on the published data [3,6]. Hong et al. reported that patients with infantile LS manifest delayed development, motor weakness, and ataxia, and patients with late-onset LS present with vertical gaze palsy and motor weakness and ataxia. However, birth history, family history, system organ involvement, and time interval from the first clinical manifestation to LS diagnosis were not statistically significant [11]. Sakushima et al. proposed the diagnostic criteria for LS in 2011, tabulated in Table 3 [12].

**Table 3:** Proposed diagnostic criteria for Leigh syndrome. CT: computed tomography, MRI: magnetic resonance imaging, CSF: cerebrospinal fluid Source: [12]

Parameter	Findings
Clinical history and physical examination	Failure to thrive, mental retardation, pyramidal signs, ophthalmoplegia, dysarthria, deafness, or other neurological manifestations
Imaging (CT, MRI)	Lesions in bilateral basal ganglia, or brain stem,
Biochemical parameter	Elevated serum or CSF lactate level
Muscle biopsy	Mitochondrial abnormalities, mitochondrial gene mutations
Rule out	Multiple sclerosis, infections, metabolic disorders, toxins, and Wernicke’s encephalopathy

Our patient presented with signs and symptoms consistent with the clinical, biochemical, and radiological criteria for LS. Muscle biopsy also reinforced the diagnosis of LS with typical imaging findings on imaging modalities. Our patient responded well to the treatment, similar to the case reported by Goldenberg and his colleagues [4].

## Conclusions

Although LS typically manifests in childhood or infancy, adult-onset LS is a rare variant, with onset mainly occurring in the second or third decade of life. Diagnosis of late-onset LS exhibits significant challenges due to its diverse clinical manifestations and overlapping features with other neurological syndromes. Compared with infantile LS, late-onset LS has a favorable prognosis. Our case highlights the importance of timely recognition, genetic testing, multidisciplinary care, and individualized treatment in improving the clinical outcomes and quality of life for LS patients.
